# Roadmap for low-carbon ultra-low temperature storage in biobanking

**DOI:** 10.1186/s12967-024-05383-5

**Published:** 2024-08-08

**Authors:** Matthew Graham, Gabrielle Samuel, Martin Farley

**Affiliations:** 1 Department for Global Health and Social Medicine, King’s College London, London, WC2R 2LS UK; 2 Medical Research Council, Swindon, SN2 1FL UK

**Keywords:** Biobanking, Sustainability, Ultra-low temperature freezers, Liquid nitrogen storage, Carbon footprint

## Abstract

Biobanks have become an integral part of health and bioscience research. However, the ultra-low temperature (ULT) storage methods that biobanks employ [ULT freezers and liquid nitrogen (LN2)] are associated with carbon emissions that contribute to anthropogenic climate change. This paper aims to provide a ‘Roadmap’ for reducing carbon emissions associated with ULT storage in biobanking. The Roadmap offers recommendations associated with nine areas of ULT storage practice: four relating to ULT freezers, three associated with LN2 storage, and two generalised discussions regarding biosample management and centralisation. For each practice, we describe (a) the best approaches to mitigate carbon emissions, (b) explore barriers associated with hindering their implementation, and (c) make a series of recommendations that can help biobank stakeholders overcome these barriers. The recommendations were the output of a one year, UK-based, multidisciplinary research project that involved a quantitative Carbon Footprinting Assessment of the emissions associated with 1 year of ULT storage (for both freezers and LN2) at four different case study sites; as well as two follow up stakeholder workshops to qualitatively explore UK biobank stakeholder perceptions, views, and experiences on how to consider such assessments within the broader social, political, financial, technical, and cultural contexts of biobanking.

## Introduction

Biobanks collect and process a wide variety of biological samples and associated data. They provide a crucial role in biomedical, health-oriented research because, by providing researchers access to these samples and data [1], they decrease the time and resources researchers would otherwise spend on collecting, storing and curating their own samples [2]. Biobanks vary in size, from small scale project-led repositories, to university or institutional biobanks, to national and international institutions, though the drive for ever larger biobanks continues, with some of the biggest biobanks collecting samples and data from a million or more patients and/or participants [3, 4].

While biobanks have become integral to health research, they are associated with adverse environmental impacts (Text A in Appendix 1). Most prominently, this relates to the carbon emissions associated with the ultra-low temperature (ULT) storage of biobanked biosamples. Ultra-low temperatures—typically considered temperatures below the − 30  °C ordinarily reached by standard freezers—are normally achieved using ULT freezers and/or liquid nitrogen (LN2), using large amounts of energy [5]. ULT freezers are the more ubiquitous storage method of the two, with each freezer sometimes consuming as much as 20 kWh per day, more than the average daily energy consumption of a UK household [6, 7].

Given the increasing risk that anthropogenic climate change poses to both humanity and the planet as a whole, as well as increasing societal concern about environmental issues more generally [8], it is imperative that stakeholders within biobanking and the medical and life sciences research field as a whole both understand the global warming contribution and other environmental impacts associated with ULT storage, as well as take steps to mitigate these impacts as much as possible. While some research regarding the environmental impacts of ULT storage exists, both in terms of carbon specific impact and wider impacts [9–13], there has been minimal drive to connect this research to a greater understanding of how this should be considered within biobanks *in practice*. Indeed, the idea that the biobanking sector should consider its own adverse environmental impacts at all represents a relatively new shift [14]. In line with this thinking, the environment needs to be considered alongside other pillars of biobanking sustainability, that is, financial and social sustainability. At the same time, how to implement such a perspective in practice raises concerns, with recent research suggesting that this is often difficult for those who manage and use biobanks because their responsibilities are often entangled with the cultural milieus and institutional networks to which they belong [15].

To address this, the aim of this paper is to provide a ‘Roadmap’ for how an environmental perspective can be implemented in practice in one specific aspect of biobanking: reducing carbon emissions associated with ULT storage (this paper is primarily focused on UK biobanks, however we believe the lessons can be applied globally) (Text B in Appendix 1). This Roadmap represents the output of a one year, UK-based, multidisciplinary research project that involved a quantitative Carbon Footprinting Assessment (CFA) of the emissions associated with one year of ULT storage [for both freezers and LN2 (reported in more detail in a forthcoming paper)] at four different case study sites (see Text C in Appendix 1 for full list), and two follow up stakeholder workshops to qualitatively explore UK biobank stakeholder perceptions, views, and experiences on how to consider such assessments within the broader social, political, financial, technical, and cultural contexts of biobanking (see Text D in Appendix 1 for methodology). Our roadmap provides a series of recommendations for nine important areas of practice relevant to mitigating the carbon emissions associated with ULT storage: four specific to ULT freezers, three for LN2 storage, and two generalised discussions regarding sample management/security and centralisation. These are listed below (Table 1). There are no simple solutions for implementing these nine practices, and as such, our recommendations reflect a balance between understanding the importance of each practice for reducing carbon emissions associated with ULT storage, alongside the current barriers associated with their implementation. The remainder of the paper discusses these practices in detail.

**Table 1:** Nine ULT storage interventions, broken down by relevant area of practice

Relevant biobanking practice	Intervention
Ultra-low temperature freezers:	1. ‘Warming up’ ULT freezers:
	2. ULT freezer management practices and cooling strategies
	3. Thorough assessment of ULT replacement strategies
	4. End-of-life practices:
Liquid nitrogen	1. LN2 delivery and manual refill
	2. Auto-refill vs. manual refill
	3. On-site generation
Sample management/security and centralisation:	1. Effective sample management and security
	2. Centralisation

## Ultra-low temperature freezers

### ‘Warming up’ ULT freezers

**Fig. 1:**
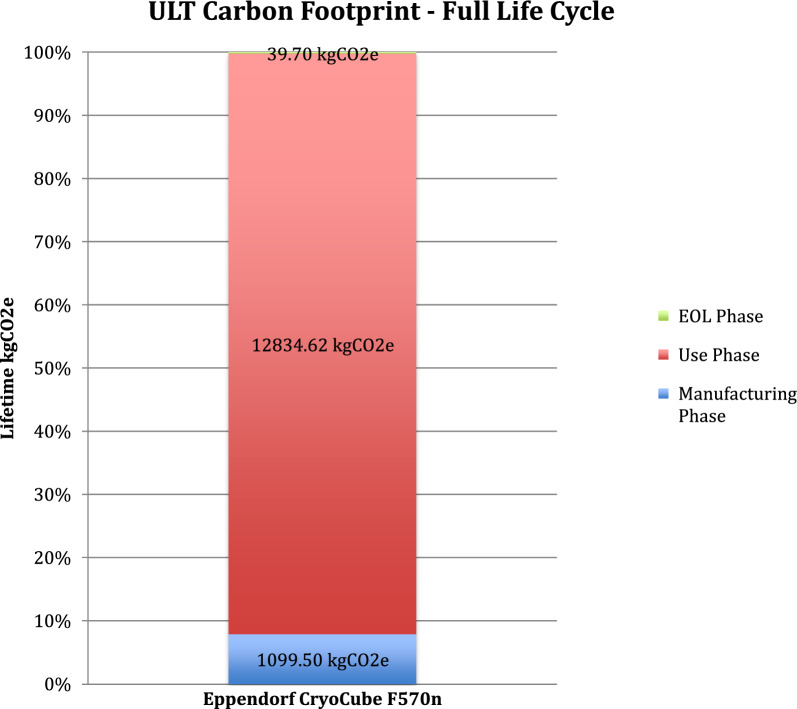
The lifetime kgCO_2_e impact of an Eppendorf CryoCube F570 n—see “Text F” for more detail

The massive energy savings that can be achieved by warming up the temperature during a ULT freezer's use phase makes it a crucial intervention to prioritise when attempting to reduce energy consumption of ULT storage. Raising ULT freezers from − 80** °**C to − 75 °C has been shown to reduce electricity consumption by 15%, with that figure rising to 28% when a ULT is warmed 10 °C to − 70 °C [11], as well as prolonging a ULT freezer’s life by reducing the stress placed on the compressor over the course of its life cycle. Moreover, for ULT freezers that are housed in rooms with heating, ventilation, and air conditioning (HVAC) systems, the freezer requires less energy to maintain lower internal temperatures, therefore expelling less heat and, in turn, lowering HVAC requirements to maintain room set-temperature. This provides a large carbon saving for ULT storage given that the results of our own Carbon Footprinting Assessment, as well as other studies assessing the carbon impact of ULT freezer life cycles, show that the energy consumed during the use phase of a ULT freezer’s life cycle accounts for upwards of 90% of the product’s entire carbon footprint (assuming a standard UK electricity grid mix), or just under 13 tonnes of CO2e (Text E, Text F., Fig. 1 in Appendix 1, [9, 13]). To put this in perspective, the average household fridge-freezer’s electricity consumption will account for 0.89 tonnes of CO2e in the same time frame (12 years) [16].

Given the potential energy savings achieved from warming up, it has long been advocated as an important strategy for reducing carbon emissions [17–20], with advocates also pointing to the mutual financial benefit due to the money saved on energy bills. Nevertheless, a norm has developed within biobanking, and laboratory science more generally, to maintain ULT freezers at − 80 °C as default [11, 20]. This norm has become so widespread that within biobanking culture there is now a strong resistance to warming ULT freezers above this temperature. In fact, many biobank managers have raised concerns about effects on sample quality and viability at higher temperatures—concerns that some authors argue are blurred by emotional attachments to the samples themselves, with reports of biobank managers labelling them as “precious” and “irreplaceable” [15].

Concerns about sample quality are underpinned by the ‘magic number’ of cryopreservation of − 136.5 °C—a temperature known as the ‘glass transition stage of water’ [21]—at which the vast majority of metabolic activity ceases. At this temperature, sample quality and viability is perceived to be ensured for long periods of time [5], with sample quality being more ‘secure’ the closer the temperature is to − 136.5 °C. Once technical capacity permitted ULT freezers to operate at − 80 °C, this became deemed the *optimum* temperature—possibly also because the number corresponds to the sublimation point of ‘dry ice’ (− 78.5 °C), a fact that seems to have contributed to historical standards in cryopreservation [22, 23].

Some scholars have criticised the importance of the − 80 °C set-temperature, arguing that it merely represents a push from ULT manufacturers as they invested in evermore technological capacity as a way to sell newer models capable of reaching ever colder temperatures, rather than the fact that these lower temperatures were *necessarily* required for the maintenance of biosample quality [17, 19]. While these claims have not been substantiated, prior to the turn of the century, ULT freezers operated at − 70 °C without any known effect on sample quality [22, 24]. Furthermore, a growing body of evidence points toward ongoing safety and stability of biosamples stored at − 70 °C [19].

Nevertheless, despite the progressing demystification of the − 80 °C figure, biobank managers have other concerns to contend with. Research suggests that the internal temperature of ULT freezers can often fail to correlate with the display temperature [25] meaning that a − 80 °C display temperature could mean an internal temperature of several degrees higher than − 80 °C. During our workshops, participants explained that this was a cause of concern for some, as a − 70 °C display temperature might actually mean an internal temperature closer to − 65 °C.

Logistical issues also contribute to reluctance to warm up. ULT’s can often hold more than one researcher’s samples, leading to a collective reluctance, either on the side of technical staff or researchers (or both), due to the perceived risk of jeopardizing multiple research projects. Furthermore, some research projects may have started storing samples at − 80 °C and while a researcher may be willing to warm up in principle, there is a fear that it might impact the reproducibility of their experiments.

Workshop participants also raised concerns that funding bodies stipulate that ULTs used for storage in research projects must be set to − 80 °C. Furthermore, they spoke about the need to align with the regulatory requirements of the UK Human Tissue Authority (HTA), which licenses biobanks. Participants emphasised that Designated Individuals (DI) (the individual within the biobank designated to ensure regulatory compliance [26]) are uncertain about how the HTA would react should a DI choose to warm up a biobank’s ULTs, and therefore are concerned that DIs would bear the burden of negative repercussions. Finally, participants explained that those who worked at larger biobanks, which collect samples for access by a range of researchers for unspecified future purposes, may be particularly cautious about warming up freezers because of the uncertainty about the effect on the full range of (potentially currently unknown) analytes within samples that future researchers might seek to access.

#### Recommendations

- To demystify the − 80 °C norm that currently exists in biobanking, we recommend:
  - stakeholders to refrain from using ‘− 80s’ as a shorthand for ULT freezers as this perpetuates the − 80 °C norm.
  - widespread promotion of the University of Colorado, Boulder database [19], which records instances of active and successful − 70 °C ULT set point temperature practice.
  - promote future research on the quality and viability of common analytes stored at − 70 °C vs. − 80 °C in order to assuage biobank stakeholder concerns.
  - compiling historic and future research and evidence that specifically explores the effects of − 70 °C on samples into an accessible comprehensive database/library.
  - intra-institutional promotion of warming up examples—workshop participants emphasised that once researchers saw that freezers within a biobank had been warmed up without any effect on sample quality and viability, this practice ‘rippled out’ and encouraged more to follow suit.
  - implementing warming up protocols at the start of a research cycle, when researchers can be confident that it will not have an effect on the reproducibility of their experiments.
  - biobank managers supporting researchers to warm up ULT freezers by referencing existing evidence of sample viability at -70 °C, as well as the potential financial and carbon-saving benefits—as one of our workshop attendees put it—“don’t mandate researchers, take them on a journey”.
  - backup ULT freezers, which are maintained in case of emergencies, such as freezer failure, should be maintained at higher set point temperatures than in use ULTs [11] and be filled with materials such as polystyrene or spare ULT racking to increase thermal mass inside the freezer (see ULT freezer management practices and cooling strategies for more information).
  - deconstructing attitudes towards sample preciousness—(unpacked further in Sample management and centralisation).
- Biobank managers should be aware of discrepancies between ULT display temperatures and internal temperatures, which could be redressed by requesting extra temperature probes from manufacturers.
- Biobank stakeholders should push manufacturers to address internal temperature issues and guarantee the proper functioning of new ULT models.
- Biobank stakeholders should push for clarity from HTA and funding bodies regarding their stance on warming up in order to ease DI concerns. This process can begin with researchers requesting specification in the early stages of HTA applications.

### ULT freezer management practices and cooling strategies

Effective ULT management best practices to help maintain biosample quality can ensure the efficient running of freezers and therefore play a vital role in decreasing freezer-associated energy use. These include regular de-icing, regular cleaning of air filters, efficient use of freezer space and capacity, maintenance of an appropriate room set point temperature, adequately spaced freezers, and limiting the number of daily door openings. They also include the location of ULT freezers: institutional building space constraints often lead to the placement of ULT freezers in unusual locations such as corridors or basements, which lack proper ventilation and/or air conditioning. This leaves them vulnerable to excess heat build-up (due to heat expelled by ULT freezers’ compressors, external weather conditions, or both) which, in turn, affects freezer efficiency [10]. As a result, most facilities will require a cooling strategy. Both passive and active solutions are possible, including the positioning of freezers so that heat is not trapped but pushed out, as well as the usage of supplementary cooling.

The importance of these practices for reducing the energy consumption of ULTs is often not stressed enough. Combined dust build-up on the filter and an obstructed door seal from a lack of de-icing can increase energy consumption by as much as 27% [10, 25]—an energy loss that approximately equates to the gains associated with warming a ULT freezer by 10 °C. In another example, poor door-opening practices (leaving doors open for longer than needed) can lead to a rise in a freezer’s internal temperature [7, 11], which forces the compressor to work harder, using more energy, and ultimately placing increased stress on the compressor over time [10]. Meanwhile, poor ULT freezer placement can lead to a 4% increase in energy consumption [25].

The stresses placed on ULT freezer’s compressors also becomes relevant when considering biobanks’ need for backup freezers. These freezers will, by definition, be mostly empty, and ULT freezers with little to no thermal mass inside will require the compressor to work harder in order to maintain set point temperature [27]. As mentioned in Sect. "‘Warming up’ ULT Freezers", backup freezers should maintain a higher set-point temperature in order to save energy and reduce compressor load, but they might also be used as decanting spaces when in use freezers are being defrosted, so that this compressor wear might be spread across a biobank’s ULT catalogue.

#### Recommendations

- A best practice freezer management plan should be implemented to ensure all freezers are working as close to their optimal efficiency as possible and that energy savings can be secured.
- Backup ULT freezers can be used as decanting spaces when fully loaded ULTs are being defrosted in order to share the load on ULT compressors across a biobank’s ULT catalogue.
- Biobanks should employ a facility cooling strategy:
  - effective spacing between ULT freezers.
  - maintain a facility temperature of between 15 and 20 °C, through both passive and active cooling solutions [28, 29].
- Biobank managers need to maintain and document freezer management programmes to ensure that ULT freezer replacement is due to freezer inefficiency caused by age and/or mechanical fault, rather than poor management practices.

### Thorough assessment of ULT replacement strategies

Our Carbon Footprinting Assessment of ULT freezers, and other such analyses, suggest that because the energy generated by a ULT freezer’s use phase dwarfs that generated by its manufacturing phase ([9, 13, Text F, Fig. 1 in Appendix 1), within most scenarios it makes sense *solely* from a carbon perspective to replace ULT freezers even if they are relatively new and their energy efficiency has only dropped slightly. This is because the amount of electricity saved by replacing a relatively young freezer with a new more efficient replacement will always outweigh the amount of carbon ‘lost’ by not using the old unit for its full lifespan, despite the replacement only running at a marginally better efficiency. For example, if we take 7.5 kWh per day as an assumed ‘maximum’ efficiency for a new replacement ULT (570 L), a figure cited in manufacturer’s literature [30], if a biobank manager meters their freezers and finds that a 5-year old freezer is running at 10 kWh per day, it would make ‘carbon sense’ to replace it.

However, this distorted picture ignores two important factors. First, freezer replacement is expensive and biobanks often lack resources. Whilst replacement is often touted as a cost savings method because of savings in energy use, only in extreme cases does replacement become justified from a financial perspective (such as when a freezer is performing at two to three times the energy efficiencies that a replacement would) (Text G, Table 2 in Appendix 1). Second, as we saw above, new ULT freezers may not operate at ‘maximum’ efficiency in practice because of poor freezer management practices. Equally, older freezers may run at high efficiencies even though it is generally assumed that they decrease in efficiency as they age up to their end of ‘lifespan’ (normally placed at somewhere between 10 and 12 years). In fact, during our research, we came across freezers that maintained efficiency levels comparable to ‘new’ freezers well-over the 12-year mark (Table 3 in Appendix 1), and some workshop participants discussed freezers running efficiently upwards of 20 years, suggesting that hypothetical energy efficiency claims can be inaccurate in practice. Indeed, in the same vein as the − 80 °C debate, a pertinent question emerges regarding the origin of the 10–12 year lifespan figure, though not one we have to the time to explore here.

As such, a replacement strategy based on hypothetical maximum efficiencies of ULTs is fundamentally flawed. Instead, collecting ‘in situ’ data on ULT freezers through freezer metering is needed—this is despite the financial and time-based considerations associated with the increased workload it entails for biobank employees. Without this metering data, a replacement strategy can only be based on the limited assumption that new freezers operate at their maximum efficiency, and older freezers operate far less inefficiently, without ruling out the possibility that poor ULT management practices are contributing to energy inefficiencies.

#### Recommendations

- Rather than a blanket policy of age-based replacement, we recommend that replacement should occur only when metering data can be collected. Replacement of functional freezer units should only be considered where there is a potential energy saving of 2.5 kWh per day available.
- Where the above is not possible, emphasis should be placed on *why* a freezer is not performing and an understanding of a biobank site’s limitations and areas of potential improvement, before replacement is considered (also see section below).
- If these conditions are met and ULT is still not performing as well as a new ULT might be expected, then replacement should be considered, targeting the oldest units first.

#### End-of-life practices

End-of-life (EOL) best practice for ULT freezers is a relatively unexplored research area, however, in principle, they are similar to best practices in EOL scenarios for domestic refrigeration appliances. ULT freezer removal and disposal services must perform relevant processes to minimise emissions, including ensuring that oil and refrigerants from the ULT’s compressor are drained so that gasses with high global warming potentials can be removed and degassed. This is particularly important for older ULT models that utilise hydrofluorocarbon (HFC) refrigerants, such as R-404a and R-508b, which have global warming potentials of 4728 and 13,412 times that of carbon dioxide, respectively [8]. Should large quantities of these gasses escape the ULT system due to improper disposal it could multiply the carbon footprint of the ULT’s lifecycle by many times. This point equally applies to the processing of polyurethane foam used for insulation in older ULT freezer models: older ULT freezers used HFC blowing agents in the manufacturing of polyurethane foam, such as HFC-245fa, which has a global warming potential of 962 times that of carbon dioxide [8, 31].

Beyond gas escape, waste management centres should reclaim recyclable material, such as steel, from the ULT unit, to ensure that the EOL phase has as low an impact on a ULT freezer’s overall carbon footprint as possible. Finally, if a ULT freezer is being replaced due to energy concerns, rather than a complete age-related failure, biobanks might consider recycling their ULT unit with a company that refurbishes second-hand laboratory equipment for re-use [32]. This can extend the overall lifespan of a ULT freezer by allowing a second-hand user to avoid incurring the full carbon price of a newly manufactured ULT freezer, provided the second-hand freezer runs at comparable efficiency to that of a new ULT.

#### Recommendations

- Ensure the use of waste management services that provide explicit information on their disposal processes when disposing ULT freezers.
- During tender for new units or when units are to be disposed of, biobank managers should engage manufacturers and suppliers to reclaim parts and materials.
- Consider repurposing ULT freezers through laboratory equipment reclamation companies, unless freezer units have been identified as particularly inefficient, in which case they should be targeted for disposal.

## Liquid nitrogen

Very little research has assessed the carbon footprint of liquid nitrogen (LN2) storage, and as such, the literature regarding best practices for LN2 storage in biobanking is almost entirely focused on safety as opposed to considerations around carbon impact. This focus is appropriate given the danger LN2 handling poses, but is also indicative of a lack of understanding and knowledge surrounding its associated carbon impact. This was reinforced by our workshop participants who noted that the carbon impact of LN2 is neither discussed in their respective biobanks, nor did they know how to reduce carbon emissions associated with the use of LN2 storage. In the following sections we explore the carbon footprint of LN2 storage, discuss strategies to mitigate this footprint, whilst also surveying the barriers that prevent low-carbon LN2 storage in biobanking.

### LN2 delivery and manual refill

**Fig. 2:**
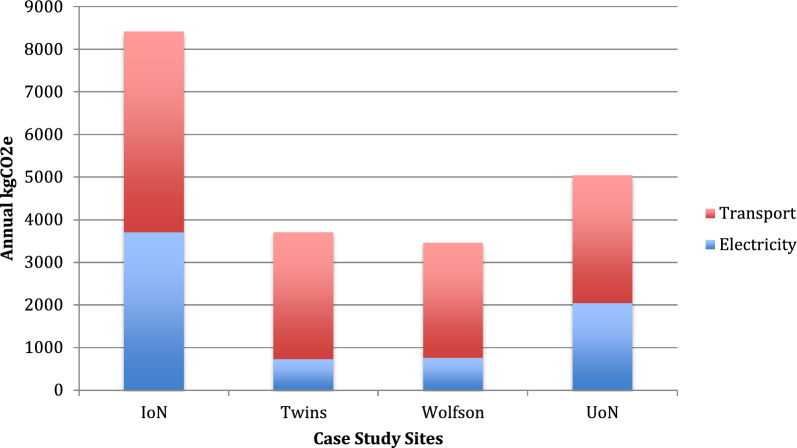
A comparison contributions of electricity use and road transportation of the carbon footprint of respective case study sites

For all our case study sites, road emissions associated with delivery from industrial manufacturing sites to respective use sites accounted for at least half of the carbon footprint associated with LN2 storage, ranging from a low of 56% to a high of 80% total carbon emissions (Text H, Fig. 2 in Appendix 1). Furthermore, all our case study sites had negligible differences in distances between manufacturing sites, distribution hubs, and use sites because of their proximity to major cities, meaning that these figures might be even larger for those biobanks further from distribution hubs. Factors affecting variation in total carbon emissions included the volume of LN2 being delivered each year, the frequency of delivery, and the distance between manufacturing sites, distribution sites and case study sites. For all respective case study sites, the smaller the volume of LN2 delivered per annum and the more frequent LN2 deliveries were per year, the larger the proportion of road-associated carbon emissions. This means that for larger sites, requiring more LN2 per delivery, the emissions due to road miles are ‘spread out’ across the larger volumes being delivered. Essentially, economies of scale play a factor in LN2 storage, pointing to the relevance of centralisation as a way to mitigate road-related emissions (see “Centralisation” section below).

Given the economies of scale available, the consolidation of LN2 deliveries into larger orders suggests itself as a natural solution. This could be accommodated by the use of bulk LN2 storage tanks that allow for the storage of larger amounts of liquid nitrogen on biobanking sites. Furthermore, the use of piping to deliver LN2 directly to use points can remove unnecessary losses of LN2 when decanting into smaller vessels for transportation from storage locations. However, the storage of larger amounts of LN2 comes with a variety of practical concerns. In particular, LN2 storage requires specific safety requirements to prevent against the risk of asphyxiation: when storing LN2 in contained spaces, there is a possibility that nitrogen could build up in the air, displacing the oxygen in a room [33]. As such, large quantities of LN2 need to be stored in rooms with adequate ventilation or outside. This may create logistical issues in practice, for example, if laboratories are located on the fifth floor of a building and LN2 storage is outside due to space constraints, biobank managers will need to transport LN2 to the fifth floor when required, a task that in itself has strict safety requirements and may prove difficult or impractical [33]. The specific layout of biobanking sites might also pose difficulties when considering piping LN2 directly to use points, as seen in the example above. Furthermore, for sites that have not been set up with LN2 piping capabilities, installing piping may prove costly. However, LN2 piping systems should be considered when building purpose-built biobanking facilities (see “Centralisation” section below).

#### Recommendations

- Where the constraints considered above allow, biobanks should consolidate LN2 deliveries in order to avoid delivery multi-runs.

### Auto-refill systems

One of our case study sites—the University of Nottingham (UoN) Cell Bank—used an LN2 auto-refill system rather than the manual fill LN2 dewars that were used at our other case sites. Our analysis showed a plummet in the amount of LN2 required for this site per year with respect to storage capacity, compared to other case study sites. Specifically, the volume of LN2 required per annum per litre of LN2 storage capacity for sites that used manual refill dewars was between 13 and 15 L, compared to ~ 2 L at UoN Cell Bank (Table 4 in Appendix 1). We hypothesise that this difference is likely attributed to the loss rate associated with manual fill LN2 dewars compared to auto-refill systems. Moreover, the large capacity of UoN’s LN2 system, in which samples are stored across just two auto-refill units, allows for less LN2 escape than configurations in which samples are stored across a large number of dewars. In fact, during the workshop, participants estimated a 30% loss rate from manual fill dewars, particularly in decentralised storage configurations, in which many individual LN2 dewars are manually replenished from onsite storage vessels and then separately accessed by different researchers across the site.

#### Recommendations

- Where constraints considered above allow, biobanks should implement an auto-refill LN2 system.

### On-site generation

**Fig. 3:**
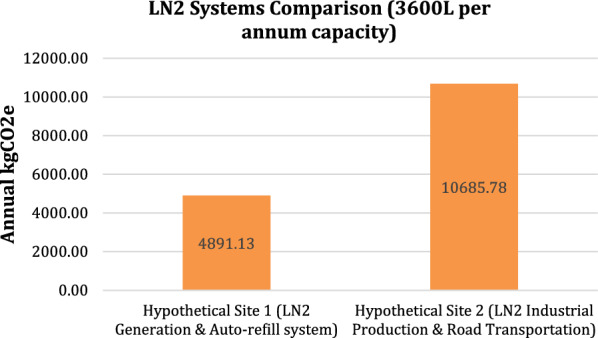
LN2 systems comparison of a hypothetical case study site using LN2 generation in combination with auto-refill system to a site using industrially produced LN2 and road transportation delivery

On-site LN2 generators are increasingly being considered for large-scale biobanks. Their major advantage versus the delivery of industrially produced LN2 is the elimination of road emissions. In fact, our analysis shows that if on-site LN2 generation is combined with auto-refill systems (described above), there is potential to reduce the LN2 storage carbon footprint by 54% (Text I, Fig. 3 in Appendix 1). On-site LN2 generation, however, is not necessarily suitable for smaller scale biobanks. LN2 generators are often prohibitively expensive, starting in the range of £20,000. Their production capacity is also far greater than required by smaller biobanks and their physical configurations can, in some instances, be prohibitive. For instance, as a part of a Higher Education Institution, the UoN Cell Bank shares its LN2 supply with a range of other laboratories, and therefore requires a large 2000 L storage vessel to be filled once weekly. The likely cost of such a large unit is upwards of £100,000; the purchase of multiple smaller units for all the laboratories relying on this supply, will be even more financially onerous. Nevertheless, for biobanks that have or are looking toward centralisating their LN2 storage, on-site LN2 generators offer a unique opportunity to drastically reduce the carbon footprint associated with their LN2 storage.

#### Recommendations

- Where constraints considered above allow, biobanks should implement an on-site LN2 generator.

## Sample management and centralisation

### Sample management and security

Sample management refers to how biobanks track, store, and utilise samples once they have been collected and processed. The number of samples stored and the time the biobank stores them will directly impact the amount of ULT storage required (both in terms of volume and time) and therefore, a biobank’s carbon footprint. From a strictly carbon perspective, this means only storing samples that are actively being used. This aligns with a biobank’s financial, institutional and ethical motivations to ensure sample usage [34–37]. However, research indicates that samples in biobanks are often underutilised [34]. Indeed, some workshop participants described storing samples that were rarely used and/or belonged to researchers that had moved institutions or projects. Here, attitudes described in the above ‘warming up’ section become relevant again, with concerns that discarding samples goes against their perceived potential future ‘value’ as ‘irreplaceable’ samples [15]. This possibility of future value is also emphasised in HTA regulation, which encourages researchers to ‘maximise’ the possibility of human tissue use before disposal [38]. The point is especially pertinent for biobanks that have been established to provide access to samples for future unknown use—as their potential value is unknown at the point of collection—but also holds true for those who have collected their own samples and stored them in institutional biobanks, because of the ‘just in case it has value’ mentality. This mindset may also manifest at the beginning of research projects, with researchers collecting more samples than are required or samples that are not strictly necessary for the research project, something one workshop participant described as ‘collection for collection’s sake.’

At the same time, underutilisation is also seen as an issue tied to the lack of infrastructure to advertise the availability of biosample collections and/or the lack of streamlined application processes for researchers to gain access to biocollections [37, 39], as well as the lack of sharing culture within biobanking. Both workshop participants and the broader literature rationalise this culture as a lack of trust and reluctance to relinquish control of samples that have taken time and effort to collect into centralised, accessible collections [15]. And while some workshop participants described increasing attention to sharing information about other biobanks’ samples with researchers, they also mentioned particular independent biobanks that were more focused on maximising financial reward and capitalising on intellectual property claims, rather than making their collections accessible for sharing.

This question of sample management also intersects with carbon emission issues when we consider the wider ramifications on sample access. While some biobanks allow open access to researchers, access at others is more controlled through designated biobank technicians and managers. While designated staff come with a cost, they provide crucial controls for the facility, and importantly, can ensure that facility storage is not abused by research staff unnecessarily maintaining large sample collections. Limited access may entail simply removing open access to the facility, but can also include providing a charge per sample (or per shelf), both to cover the cost of designated staff, but also to deter sample hoarding.

Finally, sample management involves considering which steps are in place in the event of an emergency, including which staff will be present both day and night, and who has access to biobanking protocols during those times. This is because ULT freezer failures not only represent a catastrophic possibility for researchers due to the potential for lost samples, but also the possibility of a huge carbon loss when we consider the resources already sunk into existing samples.

#### Recommendations

- If not already required by regulation, electronic management systems should be implemented in biobanks.
- If not already required by regulation, regular sample audits should be introduced in biobanks as part of sample management best practice.
- Encouragement of reflection on which samples are needed at the beginning of long-term studies, as well as the quantity of samples required—discouragement of sample collection for collection’s sake.
- ‘Use, discard, pay’ schemes that encourage researchers to either utilise existing samples, discard them, or pay for their storage on-site or in independent off-site facilities should be considered by biobank stakeholders. This could be combined with placing time-limits on sample collections at the beginning of studies, after which utilization rates will be reviewed with a view to sample discardment or payment for ongoing storage. These charges can also cover costs for designated biobanking managers.
- The storage of samples in correctly sized equipment and storage boxes to maximise storage capacity.
- Efforts should be made to bolster biobanking infrastructure within the UK. For example, the expansion of advertising infrastructure so that there is a greater awareness of which samples already exist and the formalisation of specialised biobanking networks, a possibility instantiated by the UKCRC Tissue Directory and Coordination Centre and, at a more specialised level, the UK Brain Banks Network [40, 41].
- Biobank stakeholders should engage national funders and regulators on developing clearly communicated and effective joint policy to promote the use of existing research samples and infrastructure after initial study use [42].
- An effort to change attitudes within biobanking culture should be promoted. Reframing research collections as commons, rather than ‘belonging’ to individual researchers, and encouraging communitarian approaches to biobanking, will help address over-attachment to samples and promote sample sharing amongst biobanks and researchers.
- Clear instructions should be made visually available for all staff regarding what to do in (a) the scenario of a single unit failing, and (b) a power-failure across the facility. These should include keeping units closed during power failure, unless transporting samples or adding dry-ice (the location of which should be indicated).

### Centralisation

Biobank centralisation can take different forms. For example, UK Biobank represents a large-scale, national biobanking project that collected biosamples from over 500,000 UK participants for access by many researchers [43]. Meanwhile, one of UK Biobank’s subsidiaries, the UK Biocentre, is a purpose-built biobanking facility that collects, processes, and stores huge numbers of samples on behalf of other researchers [44]. Centralisation may also entail the unification of many smaller, previously existing sample collections under a single management infrastructure [45]. Whatever approach is taken, centralisation is purported to provide financial and operational benefits from economies of scale, standardisation of biobanking practice, simplification and harmonisation of sample access, and greater quality assurance for biosamples, amongst other reasons [45–48].

Whether centralisation could also improve the carbon impact of ULT storage within biobanking remains a relatively unexplored research topic. Nevertheless, existing research, as well as workshop participants’ perceptions, indicate that some biobank stakeholders do see this potential [15]. There are many reasons to believe this view is correct. First, centralisation of ULT storage can promote standardisation of practices that reduce energy inefficiencies, such as warming up freezers, coordinated replacement and purchasing strategies, and the bulk delivery of consolidated LN2 orders. Second, the construction of purpose-built centralised facilities (where relevant) could lift pressure on existing institutional building infrastructure, a factor that often contributes to ULT freezers being stored in inappropriate locations that negatively impact energy consumption. Purpose built facilities could also allow for a building design that can mitigate safety concerns associated with LN2 storage by allowing for purpose-built ventilation systems. Third, centralisation may allow institutions to explore previously unavailable technical options, such as a Nordic system [49], that have been shown to significantly reduce the energy impact of collective ULT storage [50], LN2 piping directly to use points, on-site LN2 generators, and auto-refill systems. Centralised sample management systems might allow for more effective auditing, utilisation, and disposal protocols by streamlining the infrastructure, time, and money required for these processes, a benefit that would apply equally to LN2 and ULT freezer storage. Finally, purpose-built, centralised facilities offer the opportunity to provide additional layers of facility security, a feature that biobanks arising out of embedded institutional departments might lack.

Nevertheless, centralisation is not a silver-bullet fix. For instance, such an approach does not mean that low-carbon decision making is emphasised in operations. The UK Biocentre, for instance, runs its ULT freezers exclusively at − 80 °C. Workshop participants also shared experiences of researcher pushback to centralisation efforts based on concerns regarding their proximity to research samples (also see [15]). Where centralisation involves the construction of a new facility, space constraints will often mean that the facility must be built some distance from the existing biobank site, a fact that researchers cite as a concern, as well as being incredibly expensive. Purpose-built research facilities, large-scale ULT storage options, such as an on-site LN2 generator or a Nordic system, and centralised research infrastructures require millions of pounds of investment in order to achieve. Building such facilities also have their own embodied carbon footprint. This alone makes centralisation a long-term option for reducing the carbon impact of ULT storage that may only be available to large-scale organisations with access to vast funding networks and have calculated the net carbon emissions over a multiple-year time frame.

#### Recommendations

- We recommend the incorporation of the low-carbon case for centralisation into institutional level decision-making and planning for ULT storage:
  - for higher education institutions this process could begin with faculty restructures that allow more streamlined decision-making processes and centralised budget management. This should act as a first step before considering larger capital projects for purpose-built biobanking facilities.
  - it should also be stressed that decision-making regarding centralisation should be made in conjunction with other recommendations made in this paper:
    - before the decision to build a new biobanking facility due to space constraints, it should be a priority to ensure sample management techniques are maximising existing space and preventing expansion for its own sake.
    - old ULT storage units, both freezers and LN2 vessels, should be carried over to new facilities, *unless* a new, more efficient, large-scale system have been requisitioned i.e. Nordic system, automated LN2 system.

## Conclusion

In conclusion, mitigating the carbon emissions associated with ULT storage presents complexity. While this complexity manifests in the competing areas of interest within biobanking (financial, operational, technical, social, cultural), between a variety of different stakeholders, it also offers many areas for progress for those concerned with implementing low-carbon strategies. In our recommendations, we hope to have offered a path forward for biobanking decision-makers that are struggling to implement already well-known strategies for centering low-carbon decision making.

## Data Availability

All data generated or analysed during this study are included in this published article [and its supplementary information files]. Further data sets pertaining to the quantitative information included in the article [and its supplementary information] are available from the authors upon reasonable request.

## Appendix 1

### Text A

There is an argument that claims that biobanking as an enterprise represents a more sustainable set up to its ‘alternative’, a situation in which samples and data are not shared and many different researchers must undertake the work, and incur the environmental costs, that is done only once in a biobanking setup. This argument, however, has not been quantitatively substantiated and, moreover, is beyond the scope of this paper to address.

### Text B

#### CO_2_ vs. energy

Given the UK focus of this paper, we are working under the assumption that reducing the energy consumption of ULT storage, whether freezers or LN2, reduces the carbon footprint of these storage methods. This assumption is based on the fact that the UK currently has an emission conversion factor of 0.27386 kgCO_2_e/kWh [51], meaning that the large amounts of energy required for the ULT freezer use phase and the LN2 production phase will result in the production of CO_2_e. However, in extrapolating our recommendations to biobanks that are located in institutions/buildings that generate their own energy, as well as to different nations around the world, we understand that this assumption becomes problematic because of differences in the electrical mix. Indeed, while we did not explicitly discuss the use of renewable energy as a way of moving toward low-carbon biobanking, we do acknowledge that the transition to renewable energy sources is a crucial part of mitigating the carbon impact of ULT storage. However, it must be noted that this transition comes with its own set of environmental challenges that are beyond the scope of the paper.

### Text C

#### Case study sites

1. School of Neuroscience, Wolfson Centre, King’s College London
2. The Department for Twins Research and Epidemiology, St. Thomas’ Hospital, King’s College London
3. The Institute of Neurology, Queen’s Square House, University College London
4. The University of Nottingham Cell Bank

### Text D

#### Workshop methods

The first workshop was held online in July 2023 and attended by 14 biobank stakeholders, including researchers, biobank managers, a biobank director, and Higher Education Institution biobanking and sustainability leads. Participants were invited based on the authors’ existing network of biobanking stakeholders as well as via snowballing (n = 5 in London; n = 3 in other parts of southern England; n = 3 Midlands; n = 1 Northern England; n = 2 Wales). The workshop explored participant views on decision-making regarding ULT storage options with respect to these storage options’ environmental impact. A pre-workshop activity asked participants about their current decision-making processes in relation to low-carbon ULT storage, as well as their perspectives on which barriers and/or drivers were most prominent in their decision-making. During the workshop, we presented the results of the Carbon Footprinting Assessment on ULT storage space before holding a Q&A of open discussion in which participants shared their experiences of carbon-based decision making in biobanking. Participants then entered breakout rooms to discuss follow up questions, including:

1. When making decisions about buying a ULT freezer/LN2: Where do you get information from to make decisions and do you have enough information? Do you think the information is credible? Do the findings that we presented today change your perceptions of this information at all?
2. Are there any opportunities to overcome the barriers mentioned in the earlier discussions of this workshop/pre-workshop activity in terms of:
  - Efficient freezer management (e.g., needing to be housed appropriately)
  - Principal Investigators: how can we engage them in terms of sample management?
  - Opportunities for institutional change—how to get around issues mentioned in the pre-activity exercise (inc. power mix/renewables)?
  - Other economic/social issues that need to be overcome, and how?
  - Current regulations (are they barriers or are they useful)?
  - How can we move past the above mentioned barriers—are they insurmountable?
3. How do you imagine the future of the UK biobanking sector? What does it look like (be as broad or specific as you like)?
4. Is there anything else we need to consider in our Roadmap for low carbon ULT storage in biobanking? Is any aspect of our carbon assessment missing that needs to be included in the next workshop? Are there any other sustainability issues (environmental/waste/social/calculations that would be useful)?

The second workshop was held online in December 2023 and was attended by 7 biobank stakeholders, including four biobank managers, a sustainability manager, a researcher, and a lead of campus operations at a university (n = 3 London; n = 2 Midlands; n = 1 Northern England; n = 1 Switzerland). Two of these participants attended the first workshop, while the remaining participants were invited based on the authors’ existing network of biobanking stakeholders as well as via snowballing. The workshop explored participant views and feedback on a draft version of this paper, which was shared with participants in advance of the workshop, and ‘test’ the framework developed in the first workshop in terms of its potential efficacy and actionability.

### Text E

The information below contains text, tables and data drawn from two Carbon Footprinting Assessments (CFA) carried out by the authors as part of the project from which this paper resulted. For the purposes of this study a CFA methodology was chosen, and followed in accordance with ISO 14067:2018 standards [52]. The first CFA took an Eppendorf CryoCube F570n as its functional unit and establishes a carbon footprint for its entire lifecycle. The second CFA took 1 L of ultra-low storage (ULT) space for 1 year as its functional unit and established the carbon footprint of this unit. A full description of these studies will be presented in a forthcoming paper.

### Text F

The life cycle inventory (LCI) for the first CFA was comprised of various data categories, collected from a variety of sources. Firstly, original equipment manufacturers (OEM) were contacted in order to compile a list of components that comprise an Eppendorf F570n ULT freezer (570L listed internal volume [30]). This list was then cross-referenced with existing literature, specifically the Berchowitz and Kwon study [9]. From this list of components conversion factors were sourced from existing literature, with the Inventory of Carbon and Energy database (version 3.0) being utilized for the majority of the raw materials [53].

Existing literature was consulted in order to determine values for the raw material transformation, while OEM data was used to determine values for both the transportation of raw materials and the manufactured product to its use site. In order to determine the values associated with the product’s use phase, an average of the CryoCube F570n’s metered was taken and adjusted to account for aging over a 12-year life-span, an assumed set temperature of − 80 °C, a room set point temperature of 21 °C, used capacity of 80%, and an average of one door opening per week. As such, a power consumption of 9.7 kWh per day has been set. The emission factor for grid electricity was taken from the UK GHG Report 2023 [51]. Existing literature was consulted in order to determine refrigerant leakage rate [54]. Finally, the transportation to waste reclamation sites was calculated, and existing literature was used to gather data on the EOL processes [55].

### Text G

In considering the financial dimension of ULT freezer replacement (Table 2) we modelled a situation in which a ULT freezer with an assumed lifespan of 12 years runs at an efficiency of 8kWh per day and then modelled variables. In Table 2, it was assumed that a ULT freezer was disposed of after 8 years. We have also assumed a purchase price of £10,000 and an electricity cost of £0.31 per kWh.

### Text H

The LCI for the liquid nitrogen (LN2) portion of the second CFA was comprised of various data categories, collected from a variety of sources. Firstly, the LN2 storage capacity of each case study site was provided by the respective sites, as well as the LN2 usage per annum at each site. The energy required to produce LN2 on an industrial scale was taken from a European Industrial Gases Association position paper [56], whilst the energy required to maintain the auto-refill system at the University of Nottingham (UoN) Cell Bank was provided by the UoN. The emission factor for grid electricity was taken from the UK GHG Report 2023 [51].

To determine the contribution of road transportation to the LN2 carbon footprint, distances were calculated between case study sites, LN2 distribution hubs, and the primary sites of LN2 production. These distances were then multiplied by the emission factor for a diesel HGV (7.5–17 tonnes—rigid/average laden) provided by the UK GHG Report 2023 [51].

### Text I

The figures for the hypothetical case study site comparison of different LN2 systems were based on the figures described above (Text F), while also including figures for the electricity required to maintain an LN2 generator, which were taken from manufacturer data [57] (Figs. 1, 2, 3; Tables 2, 3, 4).

**Table 2:** Cost comparison of ULT freezer replacement—see “Text G” for more detail

ULT freezer replacement (After 8 years)		
Freezer efficiency (kWh per day)	Electricity cost saved (£)	Loss on unit (£)
10	£905.20	£3,333.33
12	£1,810.40	£3,333.33
14	£2,715.60	£3,333.33
16	£3,620.80	£3,333.33

**Table 3:** A selection of ULT freezers metered at one of the case study sites

	Freezer No.	Freezer metered	Age	Set point temperature (°C)	Room set point temperature (°C)	Capacity (L)	Door openings (monthly)	Electricity usage (kWh/day)
Case study site	1	New Brunswick—Innova U535	2 m	− 70	26.5	535	1	10.47
	2	Haier Biomedical—DW86L578J	4y	− 70	26.5	578	1	11.26
	3	New Brunswick—U570 Premium ULT Freezer	13 y	− 70	26.5	570	1	9.718
	4	New Brunswick—Innova U535	17y	− 70	21	535	1	11.87
	5	New Brunswick—U570 Premium ULT Freezer	16y	− 70	21	570	1	9.448
	6	Eppendorf CryoCube F570h	9 m	− 70	21	570	1	4.642

**Table 4:** Volume of LN2 compared to LN2 storage capacity at respective case study sites

Use site	LN2 storage capacity (L)	Volume used per annum (L)
UCL Institute of Neurology—Queen Square, London WC1N 3BG	2278	30,480
KCL Twins Research and Genetic Epidemiology—St Thomas’ Hospital Campus, Westminster Bridge Rd, London SE1 7EH	406	5967
Wolfson Centre, School of Neuroscience—Hodgkin Building, Guy’s Campus, London SE1 1UL	441	6240
University of Nottingham—Cell Bank	3600	6935

## Abbreviations

ULT: Ultra-low temperature
LN2: Liquid nitrogen
CFA: Carbon footprinting assessment
HVAC: Heating, ventilation, and air conditioning
HTA: Human tissue authority
DI: Designated individual
EOL: End-of-life
HFC: Hydrofluorocarbon
UoN: University of Nottingham
